# Piezotronic and Piezo-Phototronic Effects-Enhanced Core–Shell Structure-Based Nanowire Field-Effect Transistors

**DOI:** 10.3390/mi14071335

**Published:** 2023-06-29

**Authors:** Xiang Liu, Fangpei Li, Wenbo Peng, Quanzhe Zhu, Yangshan Li, Guodong Zheng, Hongyang Tian, Yongning He

**Affiliations:** 1 School of Microelectronics, Xi’an Jiaotong University, Xi’an 710049, China; 2 The Key Lab of Micro-Nano Electronics and System Integration of Xi’an City, Xi’an 710049, China; 3 State Key Laboratory of Solidification Processing, Key Laboratory of Radiation Detection Materials and Devices, School of Materials Science and Engineering, Northwestern Polytechnical University, Xi’an 710072, China; 4 Shaanxi Advanced Semiconductor Technology Center Co., Ltd., Xi’an 710077, China

**Keywords:** piezotronic, piezo-phototronic, core–shell structure, ZnO, heterojunction field-effect transistor

## Abstract

Piezotronic and piezo-phototronic effects have been extensively applied to modulate the performance of advanced electronics and optoelectronics. In this study, to systematically investigate the piezotronic and piezo-phototronic effects in field-effect transistors (FETs), a core–shell structure-based Si/ZnO nanowire heterojunction FET (HJFET) model was established using the finite element method. We performed a sweep analysis of several parameters of the model. The results show that the channel current increases with the channel radial thickness and channel doping concentration, while it decreases with the channel length, gate doping concentration, and gate voltage. Under a tensile strain of 0.39‰, the saturation current change rate can reach 38%. Finally, another core–shell structure-based ZnO/Si nanowire HJFET model with the same parameters was established. The simulation results show that at a compressive strain of −0.39‰, the saturation current change rate is about 18%, which is smaller than that of the Si/ZnO case. Piezoelectric potential and photogenerated electromotive force jointly regulate the carrier distribution in the channel, change the width of the channel depletion layer and the channel conductivity, and thus regulate the channel current. The research results provide a certain degree of reference for the subsequent experimental design of Zn-based HJFETs and are applicable to other kinds of FETs.

## 1. Introduction

As a third-generation electronic material, wide bandgap semiconductor materials have the advantages of high carrier mobility, strong radiation resistance, and high breakdown field strength. They have been applied in various fields such as photodetectors [1,2,3,4], surface acoustic wave devices [5,6], and solar cells [7,8,9]. Zinc oxide (ZnO) semiconductor material not only has excellent light absorption performance in the ultraviolet (UV) band due to its wide bandgap of 3.37 eV but also possesses excellent piezoelectric properties originating from its non-centrosymmetric crystal structure. Prof. Zhong Lin Wang invented the piezotronic effect in 2006, which is defined as the mechanical stimulus-induced piezoelectric polarization charges modulation on the performances of electronic devices [10]. After its invention, the piezotronic effect developed rapidly in different kinds of advanced electronic devices, with a focus on the Schottky or PN junction.

By further introducing optical excitation to the piezotronic effect, the piezoelectric and optoelectronic effects can jointly regulate the photo-excited carrier transport in semiconductors, which was invented by Prof. Zhong Lin Wang in 2010 and named the piezo-phototronic effect [11,12,13,14,15]. Using multi-effect coupling for semiconductor materials to design new electronic devices is an effective means of optimizing device performance and improving the integration of on-chip devices. By utilizing the piezotronic and piezo-phototronic effects, various high-performance electronic and optoelectronic devices can be realized [16,17,18,19,20,21]. For example, Yiyao Peng et al. prepared a self-powered flexible UV photodetector based on p-GaN/n-ZnO heterostructure, the relative optical response of which can be increased to 22% after applying −0.48% compressive strain under UV irradiation of 38.4 mW/cm^2^ [21].

Compared with a bipolar junction transistor, the channel resistance of a field-effect transistor (FET) has a negative temperature coefficient and better thermal stability. Among them, due to the lack of insulation layer capacitance, the junction FET (JFET) requires low gate driving power, a simple preparation process, and can achieve smaller device size. At present, there have been many studies on ZnO-based FETs [22,23,24,25,26,27], but most of them are the metal-oxide-semiconductor FETs (MOSFETs) or metal-semiconductor FETs (MESFETs), and there is less theoretical and experimental research on ZnO-based JFETs [28,29]. In 2019, Nan Guo et al. prepared a light-driven WSe_2_-ZnO JFET with n-ZnO nanosheets as the gate material, and the device’s optical response reached 4.83 × 10^3^ A/W, with a response time of 10 μs [29]. In addition, the tunneling field-effect transistor (TFET) as another promising candidate has been extensively studied due to its capability of lowering the subthreshold swing down to even less than 60 mV/decade [30]. Several studies in the literature have built the core–shell nanowire heterojunction structure-based TFET model and studied its characteristics, presenting superior performances compared to conventional MOSFET [31,32,33]. Moreover, bipolar junction transistors also own huge potentials in high-performance optoelectronic devices and the piezo-phototronic effect could be further utilized to optimize its characteristics [34].

Recently, our group developed a Si/ZnO thin-film-based heterojunction FET (HJFET) model (p-Si as channel) and theoretically studied its performances as modulated by the piezotronic and piezo-phototronic effects [35]. The analytical equations of Si/ZnO thin-film-based HJFET have been derived theoretically. However, during the theoretical derivation, necessary assumptions must be applied to the model so that analytical equations could be obtained. In addition, the carrier distribution inside the whole HJFET under different strain/light/electric conditions could not be mapped directly, which is not beneficial for understanding and analyzing the beneath working mechanisms.

As the size of transistors continues to decrease, in order to maintain the control of channel current by the gate, field-effect transistors have developed from traditional single gate to multi-gate to the gate-all-around structure. In this work, we establish a core–shell structure-based Si/ZnO nanowire HJFET model utilizing the finite element method. The piezotronic and piezo-phototronic effects on its performances have been systematically simulated and analyzed. The carrier distribution could be obtained and utilized to understand the working mechanism. This work helps to improve the theory of piezotronic and piezo-phototronic effects in JFETs and could be expanded to other kinds of FETs.

## 2. Simulation Model and Finite Element Method

Partial differential equations are often used to describe the state of the carriers inside a device. However, for most problems, they are difficult to solve analytically. By discretizing the partial differential equations, approximate numerical model equations are obtained, and the solutions of these numerical model equations are approximate solutions to the corresponding real solutions. The finite element method (FEM) is used to calculate these approximate solutions. All research in this paper is based on the finite element analysis software COMSOL Multiphysics.

As mentioned above, regarding the theoretical derivation of piezotronic and piezo-phototronic effects in ZnO-based HJFET, our research group has previously achieved some results [35]. Based on the previous model, here we established a core–shell structure-based Si/ZnO nanowire HJFET model using the finite element method to further verify the theoretical derivation of piezotronic and piezo-phototronic effects in ZnO-based HJFET. For the established simulation model, the p-Si core nanowire is used as the p-channel, and the n-ZnO shell is used as the gate, which is a p-channel HJFET. The n-channel HJFET has also been investigated by using the n-ZnO core nanowire as the n-channel and the p-Si shell as the gate. The parameters of Si and ZnO used in the simulation are shown in Table 1.

In classical piezoelectric theory, piezoelectric charges are often treated as surface charges because the distribution region of polarization charges is far smaller than the size of piezoelectric block materials. In our established HJFET model, we introduce the externally applied strain through the surface charge density boundary condition. The piezoelectric equation includes:

(1) $${\left(P\right)}_{i}={\left(e\right)}_{ijk}{\left(\varepsilon \right)}_{jk}$$

In the formula, P is the polarization vector/C·m^−2^; ${\left(e\right)}_{ijk}$ is the third-order piezoelectric tensor; ε is the strain tensor. According to the C_6v_ symmetry of ZnO crystal (wurtzite structure), when only the *c*-axis direction of ZnO is externally applied with strain $\varepsilon_{3}$, the corresponding polarization vector P can be obtained from Equation (1):

(2) $${P_{1}=P_{2}=0,P}_{3}=e_{33}\varepsilon_{3}$$

For ZnO piezoelectric semiconductor, $e_{33}$ is usually taken as 1.22 C·m^−2^. Assuming that the width of piezoelectric polarization charge distribution on the ZnO surface is $w_{piezo}$, then the piezoelectric charge density $N_{piezo}$ is:

(3) $$N_{piezo}=\frac{P_{3}}{qw_{piezo}}$$

At the same time, we introduce incident light illumination through a customized carrier generation boundary condition, and the generation rate of photo-excited charge carriers is related to incident light wavelength, light intensity, and material absorption coefficient. At the same time, the Shockley–Read–Hall (SRH) recombination model is added to the model, which uses the constant minority carrier lifetime shown in Table 1.

## 3. Results and Discussion

### 3.1. Basic Characteristics and Piezotronic and Piezo-Phototronic Effects on p-Channel Core–Shell Structure-Based HJFET

**Figure 1:**
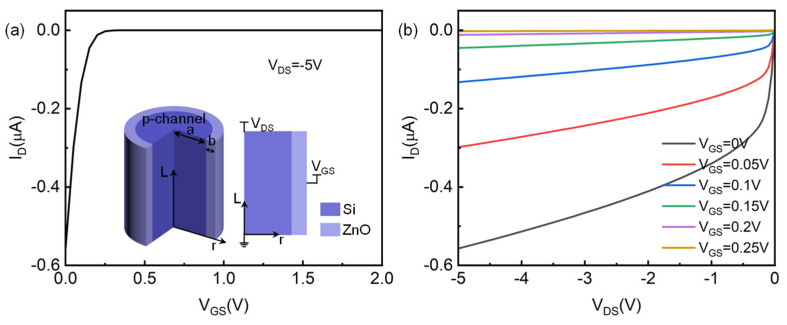
Simulation model and basic characteristics of p-channel HJFET. (**a**) Transfer characteristics. The inset shows the established model and its cross-sectional view. (**b**) Output characteristics.

Firstly, the simulation model of a p-channel core–shell structure-based HJFET is illustrated in the inset of Figure 1a. It could be seen that the radius of p-channel Si is a and the radius of whole device is a + b. The channel length is defined as L. Unless otherwise specified, the simulation parameters of the p-channel HJFET model are shown in Table 2.

The basic output characteristics of the device are shown in Figure 1. Figure 1a shows the transfer characteristics, and Figure 1b shows the output characteristics. It can be seen from the simulation results that the device indeed exhibits distinctive p-channel HJFET characteristics. At V_GS_ = 0 V, the channel is not fully depleted by n-ZnO gate and therefore current could flow in the p-channel with V_DS_ applied. With the increase in V_GS_, obviously the channel becomes fully depleted and the flowing current in the p-channel decreases. At V_GS_ = 0.3 V, the device almost turns off and the gate control effect is obvious. If we select the on-state voltage as (V_GS_ = 0 V, V_DS_ = −5 V) and the off-state voltage as (V_GS_ = 1 V, V_DS_ = 0 V), the switching ratio of the device reaches as high as 2.665 × 10^10^. The output characteristic curves under different gate voltages are also consistent with expectations. When V_GS_ = 0 V and V_DS_ = −5 V, the channel current I_D_ is about −0.557 μA. As the gate voltage increases, the channel current decreases, making it easier to reach saturation at the same drain-source voltage.

**Figure 2:**
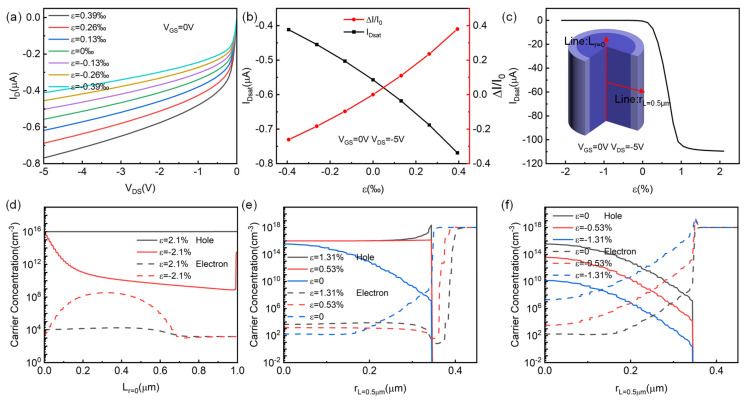
Applying strain on p-channel HJFET. (**a**) Output characteristics of HJFET after strain application. (**b**) Saturated current I_Dsat_ and its rate of change ΔI/I_0_ with applied strain. (**c**) Change of saturation current I_Dsat_ after increasing the range of applied strain. The internal illustration is the schematic diagram of the selected position. (**d**) Carrier concentration distribution at the device’s central axis under different strain conditions. (**e**) Carrier concentration distribution at the device’s central horizontal line under tensile strain. (**f**) Carrier concentration distribution at the device’s central horizontal line under compressive strain.

For the introduction of strain in p-channel HJFET, by assuming that strain induced piezoelectric charges are distributed near the core–shell Si/ZnO heterojunction interface in the area with a width $w_{piezo}$ of 1 nm, the corresponding piezoelectric charge density $N_{piezo}$ can be obtained by Equation (3). According to the theoretical calculation in the previous section, when the strain ε_3_ is 0.3‰, the strain-induced piezoelectric charge density is about 1 × 10^18^ cm^−3^ scale. Figure 2 shows the changes in the electrical properties of p-channel HJFET after introducing strain. From Figure 2a, it can be seen that after applying tensile strain (ε_3_ > 0), the channel current I_D_ increases, and after applying compressive strain (ε_3_ < 0), the channel current I_D_ decreases. The strain varies within the range of −0.39‰ to 0.39‰, achieving a monotonic saturation current I_Dsat_ increase of −0.4 μA~−0.77 μA. The corresponding saturated drain-source voltage V_Dsat_ also increases. As shown in Figure 2b, the corresponding saturation current change rate can reach 38%, where the current change rate ΔI/I_0_ is defined as (I_Dsat-ε_ − I_Dsat-ε=0_)/I_Dsat-ε=0_. Figure 2c shows the changes in the saturation current of the device when a larger strain range is applied. The direction of strain regulation remains unchanged, that is, after applying tensile strain, the channel current I_D_ increases. More importantly, when the tensile strain is increased to around 1%, the I_D_ reaches saturation with increasing tensile strain, which is about −110 μA. After applying compressive strain, the channel current change is opposite, with the I_D_ even decreasing nearly to zero. This indicates that the strain regulation itself owns a broad modulation capability of the channel current I_D_.

Figure 2d–f show the carrier concentration distribution at different positions (Line: L_r=0_ and Line: r_L=0_) of the device after the strain is applied. The schematic diagram of the selected line position is shown in the inset illustration in Figure 2c. The dashed lines in Figure 2d–f represent the electron concentration distribution, and the solid lines represent the hole concentration distribution. From Figure 2d, it can be seen that, when a large tensile strain of 2.1% is applied, the hole concentration in the channel reaches about the order of 1 × 10^16^ cm^−3^, which is close to the acceptor doping concentration in the p-Si channel. This indicates that the bias voltage of V_GS_ = 0 V and V_DS_ = −5 V cannot saturate the channel current of the device. Therefore, the saturated channel current in Figure 2c under larger tensile strain conditions will actually be higher. After applying a large compressive strain of −2.1%, the hole concentration as p-channel carriers decreases dramatically and, hence, the channel has been turned off by the externally applied compressive strain. Figure 2e,f show the carrier concentration distribution of the device’s central horizontal line under tensile and compressive strains, respectively. It can be seen from Figure 2e that after the tensile strain is applied, the width of the depletion region on the p-Si channel side decreases, while the width of the depletion region on the n-ZnO gate side increases. As a result, the p-channel carrier concentration increases, leading to an increase in the channel current I_D_. After the compression strain is applied, the change in depletion region of both sides is opposite, just as shown in Figure 2f.

**Figure 3:**
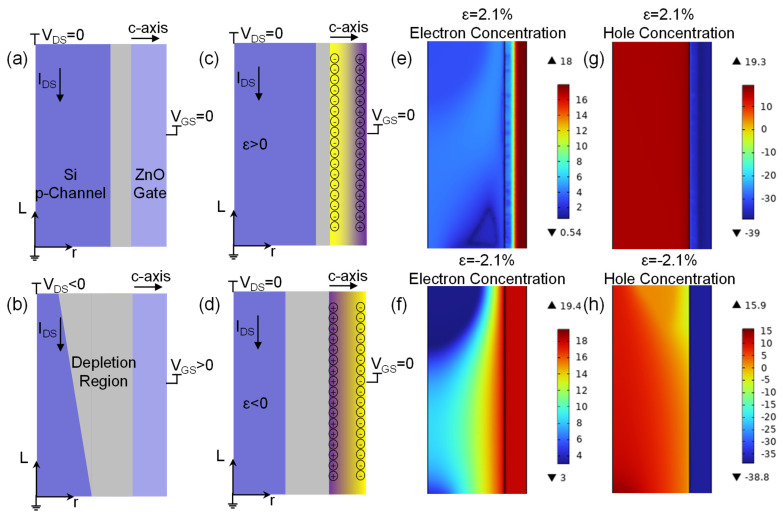
Schematic diagram of the analysis of strain regulation effect on p-channel HJFET. (**a**,**b**) Schematic diagram of p-channel HJFET under bias voltage regulation. (**c**,**d**) Schematic diagram of p-channel HJFET under tensile and compressive strain regulation. (**e**–**h**) Logarithmic distribution of carrier concentrations obtained from simulation under both bias voltage and strain regulation.

In order to analyze the regulatory effect of strain on the electrical performance of p-channel HJFET more clearly, Figure 3 provides a detailed schematic diagram of the p-channel HJFET. The *c*-axis direction of ZnO layer is always radial. Figure 3a shows the HJFET without external bias, and Figure 3b shows the HJFET controlled by bias voltage. For a p-channel HJFET, when the gate voltage V_GS_ is positive, the gate PN junction is reverse biased, the width of the depletion region increases, the conduction area of the p-channel decreases, the channel resistance increases, and, finally, the channel current decreases. At the same time, when the negative drain-source voltage V_DS_ is applied, the gate junction reverse bias voltage at the drain end is greater, thus the width of the depletion region further increases until the channel is completely depleted, that is, “channel pinch-off”. At this time, if the V_DS_ continues to increase, most of the voltage drops in the channel pinch-off area, and the channel current would no longer increase. Figure 3c,d show the strain regulated HJFET. After the tensile strain is applied, negative piezoelectric polarization charges are induced at the Si/ZnO heterostructure interface, attracting holes and repelling electrons, resulting in a decrease in the p-channel depletion region, an increase in the p-channel conduction area, and thus an increase in the channel current I_D_. The opposite modulation would be produced when the compressive strain is applied, as illustrated by Figure 3d. Figure 3e–h provides the cross-sectional views of the logarithmic distribution of charge carriers under tensile and compressive strains, which together with Figure 2e,f validate the above strain regulation working mechanism analysis.

**Figure 4:**
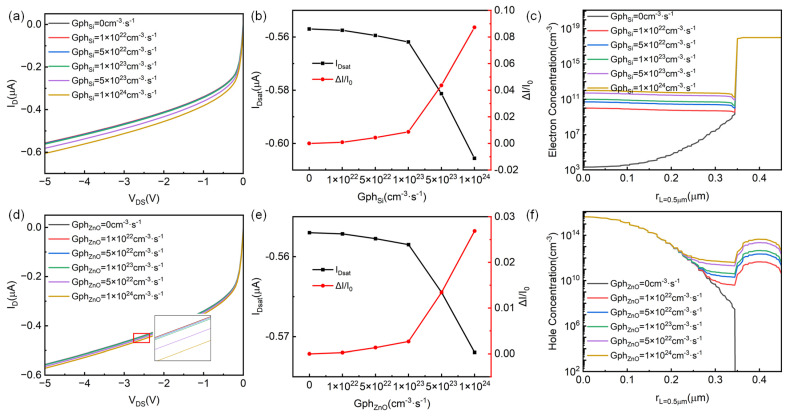
Applying light on p-channel HJFET. The output characteristics of p-channel HJFET with photo-generated carriers in (**a**) p-Si channel layer only and (**d**) gate n-ZnO layer only. (**b**,**e**) are the saturation current I_Dsat_ and its rate of change ΔI/I_0_ respectively with the photo-generated carrier production rate Gph_Si_ in Si and Gph_ZnO_ in ZnO. (**c**,**f**) are the electron concentration distribution (as minority carrier in p-Si channel) and hole concentration distribution (as minority carrier in n-ZnO gate) at the central horizontal line position of the p-channel HJFET under different light conditions, respectively.

In our established simulation model, we also introduced incident light illumination through a customized generation boundary condition to study the performance changes in the device under different light conditions, namely, only the p-Si channel layer and only the gate n-ZnO layer have photo-generated carriers. Figure 4 shows the optical response performance of the p-channel HJFET. Figure 4a,d present the corresponding output characteristic changes when the generation rate is adopted only in the p-Si channel and the n-ZnO gate, respectively. It can be seen that at the same level of photo-generated carrier generation rate, the photo-generated carriers in p-Si have a greater impact on the electrical performance of the device compared to that in n-ZnO. From Figure 4c, it can be seen that when the photo-generated carrier generation rate of Si (Gph_Si_) is 1 × 10^24^ cm^−3^·s^−1^, the minority carrier (electron) concentration in the p-channel increases to the order of 1 × 10^12^ cm^−3^, and the channel current I_D_ varies from −0.557 μA in the dark state to −0.606 μA. The saturation current change rate reaches about 9%, where the current change rate ΔI/I_0_ is defined as (I_Dsat-Gph_ − I_Dsat-Gph=0_)/I_Dsat-Gph=0_. From Figure 4f, when Gph_ZnO_ is 1 × 10^24^ cm^−3^·s^−1^, although the minority carrier (hole) concentration on the ZnO side reaches 1 × 10^14^ cm^−3^, since most of the depletion region is located in the p-Si channel, the photo-generated electromotive force is small and has little effect on device’s performance regulation. Therefore, the saturation current change rate is less than 3%. Our simulation results show that under the same carrier generation rate, the regulation of photogenerated carriers in the channel is stronger. However, in the experimental design of Si/ZnO HJFET, light intensity and wavelength are the direct factors.

**Figure 5:**
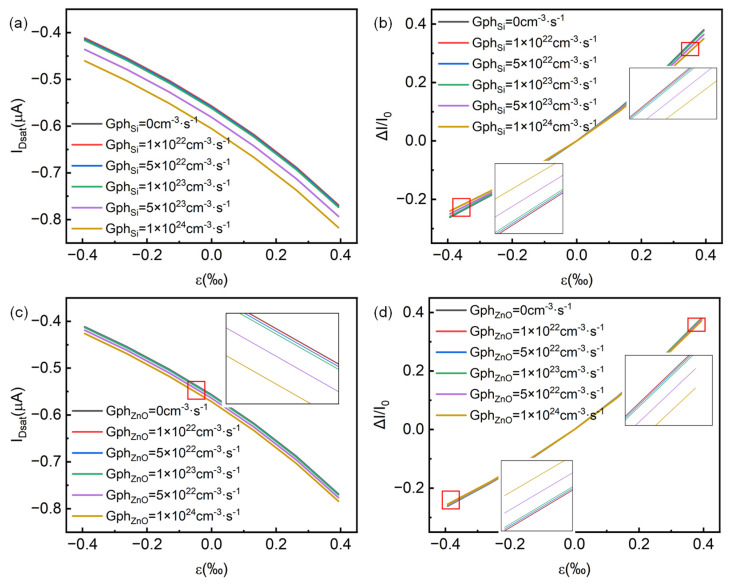
Effect of simultaneously applying strain and light on p-channel HJFET. (**a**,**b**) are the saturation current I_Dsat_ and its rate of change ΔI/I_0_ with strain ε_3_ when only the p-Si channel layer has photo-generated charge carriers. (**c**,**d**) are the saturation current I_Dsat_ and its rate of change ΔI/I_0_ with strain ε_3_ when only the gate n-ZnO layer has photo-generated carriers.

By introducing both strain and light into the p-channel HJFET, the simulation results show that the saturation channel current in HJFET varies with strain under different light conditions, as shown in Figure 5. From Figure 5a,c, it can be seen that when tensile strain is applied, the saturation channel current increases, while when compressive strain is applied, it is the opposite. Furthermore, after introducing light, the saturation channel current also increases, and the photo-generated carriers in p-Si channel have a greater regulatory effect on the device, consistent with the aforementioned phenomenon. When Gph_Si_ = 1 × 10^24^ cm^−3^·s^−1^, the saturated channel current is −0.606 μA, and the generated photocurrent is approximately 0.049 μA. From Figure 5b,d, it can be seen that, compared to the light regulation, the strain regulation effect on the HJFET is more obvious, and the saturation channel current change rate can reach a maximum of about 38%.

### 3.2. Effects of Structure Parameters, Doping Concentrations, and Gate Voltages on p-Channel Core–Shell Structure-Based HJFET

**Figure 6:**
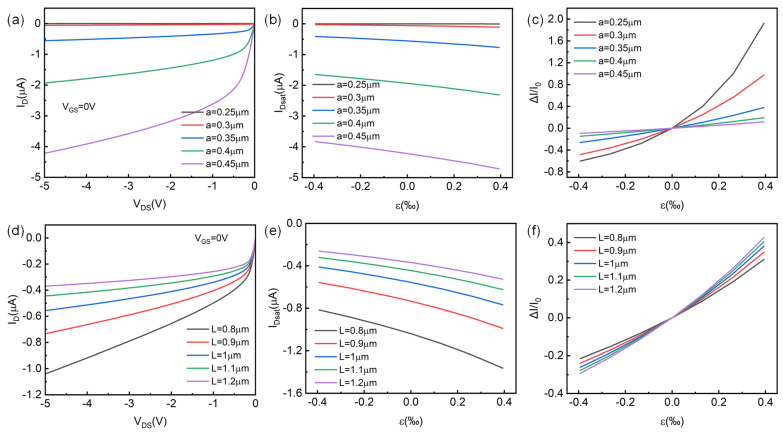
Effect of structural parameters on the piezotronic effect in p-channel HJFET. (**a**–**c**) The HJFET output characteristics, saturation current I_Dsat_, and its rate of change ΔI/I_0_ with strain ε_3_ for different p-Si channel radius a, respectively. (**d**–**f**) The HJFET output characteristics, saturation current I_Dsat_, and its rate of change ΔI/I_0_ with strain ε_3_ for different p-Si channel length L, respectively.

In this section, we conducted a scanning analysis of the structural parameters in the p-channel HJFET simulation model, and the results are shown in Figure 6. Figure 6a–c show the scanning results of p-Si channel radius a, and Figure 6d–f show the scanning results of channel length L. From the simulation results, it can be seen that the structural parameter radius a has a greater regulatory effect on device performance. When the radius a varies in the range from 0.25 μm to 0.45 μm, the saturation channel current of the device can increase from −1.31 nA to −4.218 μA. More importantly, the larger radius a, the more the saturated channel current increases when the same strain is applied, i.e., the larger ΔI. However, the saturation channel current change rate becomes smaller, as shown in Figure 6c. At a = 0.25 μm and ε_3_ = 0.39‰, ΔI/I_0_ can reach 192%. The impact of channel length L on device performance is different. The larger channel length L, the smaller the channel current. When the same strain is applied, at L = 1.2 μm and ε_3_ = 0.39‰, ΔI/I_0_ can only reach 43%. The scanning simulation of structural parameters has certain guiding significance for the experimental design of ZnO HJFET. It can be seen from the Figure 6 that the channel thickness a has the greatest influence on the channel current. Therefore, the channel thickness, that is, the Si core diameter, can be determined according to the range of the required channel current. The appropriate device length L is then selected to achieve a specific range of strain sensing.

**Figure 7:**
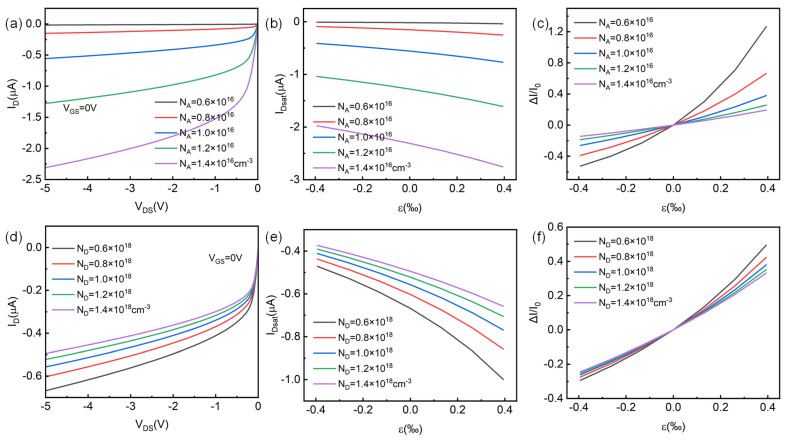
Effect of doping concentration on the piezotronic effect in p-channel HJFET. (**a**–**c**) The HJFET output characteristics, saturation current I_Dsat_, and its rate of change ΔI/I_0_ with strain ε_3_ at different acceptor doping concentrations of N_A_, respectively. (**d**–**f**) The HJFET output characteristics, saturation current I_Dsat_, and its rate of change ΔI/I_0_ with strain ε_3_ at different donor doping concentrations of N_D_, respectively.

We also conducted a scanning analysis on the doping concentration in the device, and the results are shown in Figure 7. For the convenience of comparative analysis, the parameter values of the scanned donor doping concentration N_D_ and acceptor doping concentration N_A_ are the same. From Figure 7a,d, it can be seen that the acceptor doping concentration N_A_ has a greater impact on the electrical performance of the device. When N_A_ is at 0.6 × 10^16^ cm^−3^~1.4 × 10^16^ cm^−3^, the saturation channel current of the device can increase from −17 nA to −2.3 μA. When N_D_ changes within the same range, the saturated channel current of the device only changes within the range of −0.494 μA~−0.668 μA. Furthermore, with the increase in N_A_, I_Dsat_ increases and ΔI/I_0_ decreases, whereas the impact of N_D_ on the device is different, with decreasing I_Dsat_ as N_D_ increases, but decreasing ΔI/I_0_. The channel current increases with N_A_ and decreases with N_D_. This is because when N_A_ increases, the ionized carrier concentration in the channel increases and the channel conductivity increases. At the same time, due to the electrically neutral condition, the width of the depletion layer on the channel side decreases and the channel current increases. However, when N_D_ increases, the channel conductivity remains unchanged, ignoring the influence of minority carrier electrons, but the width of the channel depletion layer increases, the channel conduction region decreases, and the channel current decreases. Compared with N_A_ modulation, N_D_ modulation only changes the width of the channel depletion layer and has less regulatory effect, so the influence of doping concentration in the channel should be mainly considered in the experimental design of ZnO HJFET.

**Figure 8:**
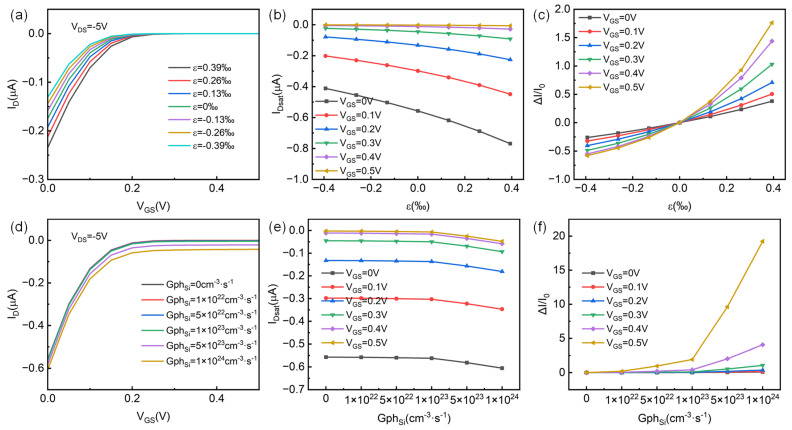
Piezo-phototronic effect in p-channel HJFET under different gate voltage regulation. (**a**) The transfer characteristics of HJFET under different strain conditions. (**b**,**c**) are the saturation current I_Dsat_ and its rate of change ΔI/I_0_ with strain ε_3_ under different gate voltage regulation, respectively. (**d**) The transfer characteristics of HJFET under different lighting conditions. (**e**,**f**) are the saturation current I_Dsat_ and its rate of change ΔI/I_0_ with photo-generated carrier generation rate Gph_Si_ under different gate voltage regulation, respectively.

In addition, electric gate voltage can also adjust the channel depletion region width in p-channel HJFET, thus adjusting the channel conductivity and channel current. This working mechanism is similar to the control of structure parameter radius a on device performance, which affects the channel current through changing the thickness of the channel conduction area in the core–shell structure. Consequently, this section mainly studies the piezo-phototronic effect in p-channel HJFET under different gate voltages. Figure 8a–c show the strain regulation effect in HJFET under different gate voltages, and Figure 8d–f show the light regulation effect in HJFET under different gate voltages, with only the case of light absorption in p-Si channel considered. From Figure 8a, it can be seen that after applying tensile strain, the channel current increases and the gate turn-off voltage increases, while applying compressive strain results in the opposite change. From Figure 8b,c, it can be seen that as the forward gate voltage increases, the saturation current of the device decreases, but the rate of saturation current change increases. These results indicate that the influence of gate voltage on the piezotronic effect in the device is consistent with the regulatory effect shown by the aforementioned structural parameter radius a. From Figure 8d–f, it can be seen that the I_D_ increases with the increase in Gph_Si_. When the forward gate voltage increases, I_D_ decreases but ΔI/I_0_ increases. At V_GS_ = 0.5 V and Gph_Si_ = 1 × 10^24^ cm^−3^·s^−1^, ΔI/I_0_ can reach a maximum of about 1924%.

### 3.3. Piezotronic and Piezo-Phototronic Effects on n-Channel Core–Shell Structure-Based HJFET

**Figure 9:**
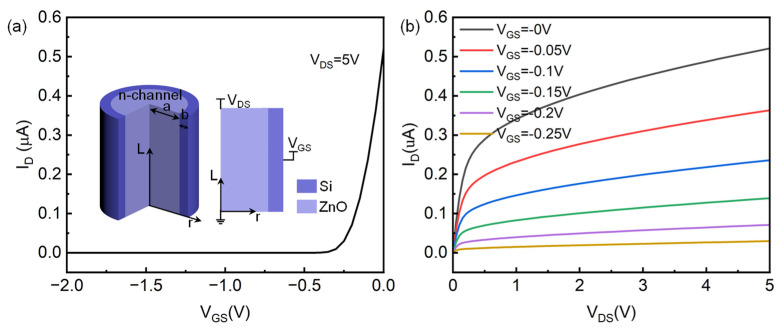
Simulation model and basic characteristics of n-channel HJFET. (**a**) The transfer characteristics of n-channel HJFET. The inset shows the simulation model and its cross-sectional view. (**b**) The output characteristics of n-channel HJFET.

Finally, the finite element method-based simulation modeling is carried out for a core–shell structure-based ZnO/Si n-channel nanowire HJFET. The model structure is shown in the inset of Figure 9a. The inner core nanowire is a n-ZnO channel, whose *c*-axis direction is pointing away from the radial direction, and the outer shell p-Si acts as the gate. If no special instructions are given, this n-channel HJFET is consistent with the model parameters of the aforementioned p-channel HJFET. In order to ensure that the depletion region is located more in the n-ZnO channel area, the donor concentration N_D_ in ZnO is adjusted to 1 × 10^16^ cm^−3^, and the acceptor concentration N_A_ in Si is 1 × 10^18^ cm^−3^. Figure 9a,b show the transfer characteristics and output characteristics, respectively. Compared with the previously studied p-channel HJFET in Figure 1, the gate turn-off voltage of the n-channel HJFET is slightly higher. When V_GS_ = −0.25 V and V_DS_ = 5 V, the device channel current is about 29.6 nA, while the p-channel HJFET only has a channel current of 2.38 nA under the same bias voltage conditions.

**Figure 10:**
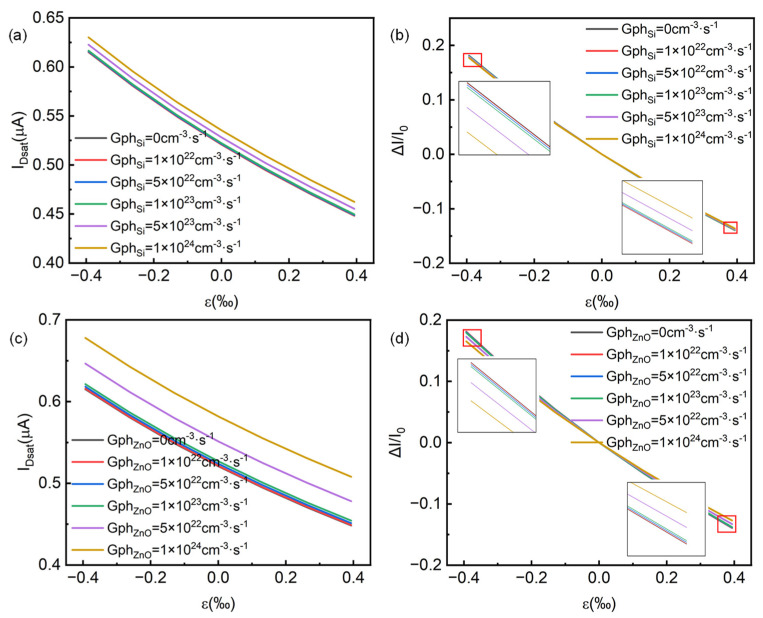
Effect of simultaneously applying strain and light on n-channel HJFET. (**a**,**b**) are the saturation current I_Dsat_ and its rate of change ΔI/I_0_ with strain ε_3_ when only p-Si gate layer has photo-generated charge carriers. (**c**,**d**) are the saturation current I_Dsat_ and its rate of change ΔI/I_0_ with strain ε_3_ when only the n-ZnO channel layer has photo-generated charge carriers.

The piezo-phototronic effect in the n-channel HJFET based on the core–shell nanowire structure has been simulated and analyzed. Figure 10 shows the changes in channel current under different strains and light illuminations. From the simulation results, it can be seen that the photo-generated carriers in n-ZnO channel have a greater regulatory effect on the device performance. When Gph_ZnO_ = 1 × 10^24^ cm^−3^·s^−1^, the saturated channel current is 0.582 μA, and the generated photocurrent is approximately 0.071 μA. When the tensile strain is applied, the channel current decreases. When the compressive strain is applied, the channel current increases, which is opposite to the strain regulation in p-channel HJFET. Taking the tensile strain as an example, the negative piezoelectric polarization charges are induced at the interface of the ZnO/Si heterojunction in the n-channel HJFET, resulting in that the channel depletion area increases and hence the channel current decreases. Moreover, the saturation channel current change rate of n-channel HJFET is much smaller. When the externally applied compressive strain ε_3_ is −0.39‰, the current change rate is only approximately 18%. These results indicate that, for the strain and light cooperated regulation, it might be better to produce the photo-excited charge carriers and the piezoelectric charges at different semiconductors. For instance, the photo-generated carriers are produced in p-Si core channel and the piezoelectric charges are produced in n-ZnO shell gate in the core–shell structure-based Si/ZnO p-channel nanowire HJFET.

## 4. Conclusions

This study mainly uses the finite element method to simulate the core–shell structure-based nanowire HJFET and studies the working mechanisms of piezotronic and piezo-phototronic effects in it. Strain is introduced through the surface charge boundary condition, and light is introduced through a user-defined generation boundary condition. Taking p-channel HJFET as an example, after applying tensile strain, the channel current increases and the saturation current change rate increases. At a tensile strain of 0.39‰, the saturation current change rate can reach 38%. The regulatory mechanism of strain on the electrical performance of HJFET has been analyzed in detail. After applying light, the channel current increases and the saturation current change rate increases. Compared with the photo-generated carriers in the n-ZnO gate, the photo-generated carriers in p-Si channel have a stronger regulatory effect on the device’s performance. However, compared to strain regulation, the effect of light regulation is much weaker. Scanning analysis has also been conducted on different parameters, including channel radius a, channel length L, doping concentrations N_A_ and N_D_, and gate voltage V_GS_. It is concluded that the channel current increases with an increase in radius a and N_A_ and decreases with an increase in channel length L, N_D_, and V_GS_. The saturation current change rate decreases with the increase in radius a, N_A_, and N_D_ and increases with the increase in channel length L and V_GS_. Finally, another n-channel HJFET model has been established and simulated to analyze its piezo-phototronic effect. It is found that under the same model parameters, the strain regulation on the saturation current change rate of an n-channel HJFET would be smaller than that of a p-channel HJFET. On the other hand, the light regulation on saturation current change rate of n-channel HJFET would be larger than that of a p-channel HJFET. This work presents that, based on the finite element method, the introduction of strain and light illumination in the simulation model is simple and facile. More importantly, the simulated parameter range of piezoelectric charge density (i.e., the strain) and the location of generation rate (i.e., the light) could be modulated conveniently, not being limited by the necessary assumptions adopted in the analytical derivations. Moreover, the carrier distribution in the whole device could be illustrated to explore the underlying device physics more clearly, providing a more precise understanding of the piezotronic and piezo-phototronic effects in FETs.

## Figures and Tables

**Table 1:** Parameters for Si and ZnO semiconductors.

Material Parameters	Si	ZnO
Relative dielectric constant	11.7	(8.5446, 8.5446, 10.204)
Electron lifetime (μs)	10	10
Hole lifetime (μs)	10	10
Band gap (eV)	1.12	3.37
Electron affinity (eV)	4.05	4.5
Valence band effective density of states (cm^−3^)	1.04 × 10^19^	3.5 × 10^18^
Conduction band effective density of states (cm^−3^)	2.8 × 10^19^	1 × 10^20^
Electron mobility (cm^2^·V^−1^·s^−1^)	1450	200
Hole mobility (cm^2^·V^−1^·s^−1^)	500	10

**Table 2:** Parameters for p-channel HJFET model.

Parameters	Value
Radius of core Si nanowire (a)	0.35 μm
Thickness of shell ZnO (b)	0.1 μm
Channel length (L)	1 μm
Acceptor doping concentration in Si (N_A_)	1 × 10^16^ cm^−3^
Donor doping concentration in ZnO (N_D_)	1 × 10^18^ cm^−3^

## Data Availability

The research data is available from the corresponding author upon reasonable request.
